# Why does rotavirus vaccine effectiveness vary around the globe?

**DOI:** 10.1371/journal.ppat.1014670

**Published:** 2026-10-09

**Authors:** Sarah Woodyear, Nabila Nawar Binti, Sarah L. Caddy

**Affiliations:** 1 Baker Institute for Animal Health, Cornell University, Ithaca, New York, United States of America; 2 Department of Microbiology and Immunology, College of Veterinary Medicine, Cornell University, Ithaca, New York, United States of America; Washington University School of Medicine in Saint Louis: Washington University in St Louis School of Medicine, UNITED STATES OF AMERICA

## Abstract

Rotavirus vaccines have transformed the prevention of severe childhood diarrhea globally, yet their effectiveness remains markedly lower in low- and middle-income countries (LMICs), where rotavirus mortality is greatest. Understanding the biological and environmental determinants of this disparity is critical for optimizing existing vaccination strategies and informing next-generation vaccine development. This review examines the factors contributing to variable rotavirus vaccine effectiveness across different settings, with a focus on maternal antibodies, infant gut health, co-infections, co-vaccinations and human genetic polymorphisms. Maternal antibodies, transferred transplacentally and through breast milk, can inhibit vaccine replication in the infant gut. Maternal antibody interference may be exacerbated in LMICs due to higher maternal antibody levels, driven by increased rates of natural rotavirus exposure and prolonged breastfeeding. Malnutrition, disruption of the gut microbiome, and environmental enteric dysfunction collectively impair intestinal immune responses, with their relative contributions also varying by geography. Co-administration of the oral poliovirus vaccine and concurrent enteric infections represent additional sources of interference that are more prevalent in resource-limited settings. Host genetic factors, particularly polymorphisms in fucosyltransferase genes governing histo-blood group antigen expression, further modulate vaccine immunogenicity across populations. These factors do not act in isolation; rather, they interact to shape the gut environment and collectively limit oral vaccine replication. Addressing rotavirus vaccine inequity will require context-specific strategies that combine optimized vaccine scheduling, nutritional and microbiome-targeted interventions, and the development of novel vaccine platforms capable of overcoming the unique immunological challenges of LMICs.

## 1. Introduction

Development and delivery of rotavirus vaccines have been fundamental in reducing severe childhood diarrhea, a leading cause of morbidity and mortality in young children worldwide [1]. Live-attenuated oral rotavirus vaccines, such as Rotarix (GSK) and RotaTeq (Merck), have been incorporated into national immunization programs in 141 countries, and have demonstrated high levels of protection against severe rotavirus-induced gastroenteritis in high-income countries (HICs) [2–4]. However, vaccine effectiveness is not consistent across regions. While rotavirus vaccines can achieve effectiveness rates of 80%–90% in wealthier settings, their performance is often significantly worse in low- and middle-income countries (LMICs), where the burden of disease is highest (Fig 1).

**Fig 1:**
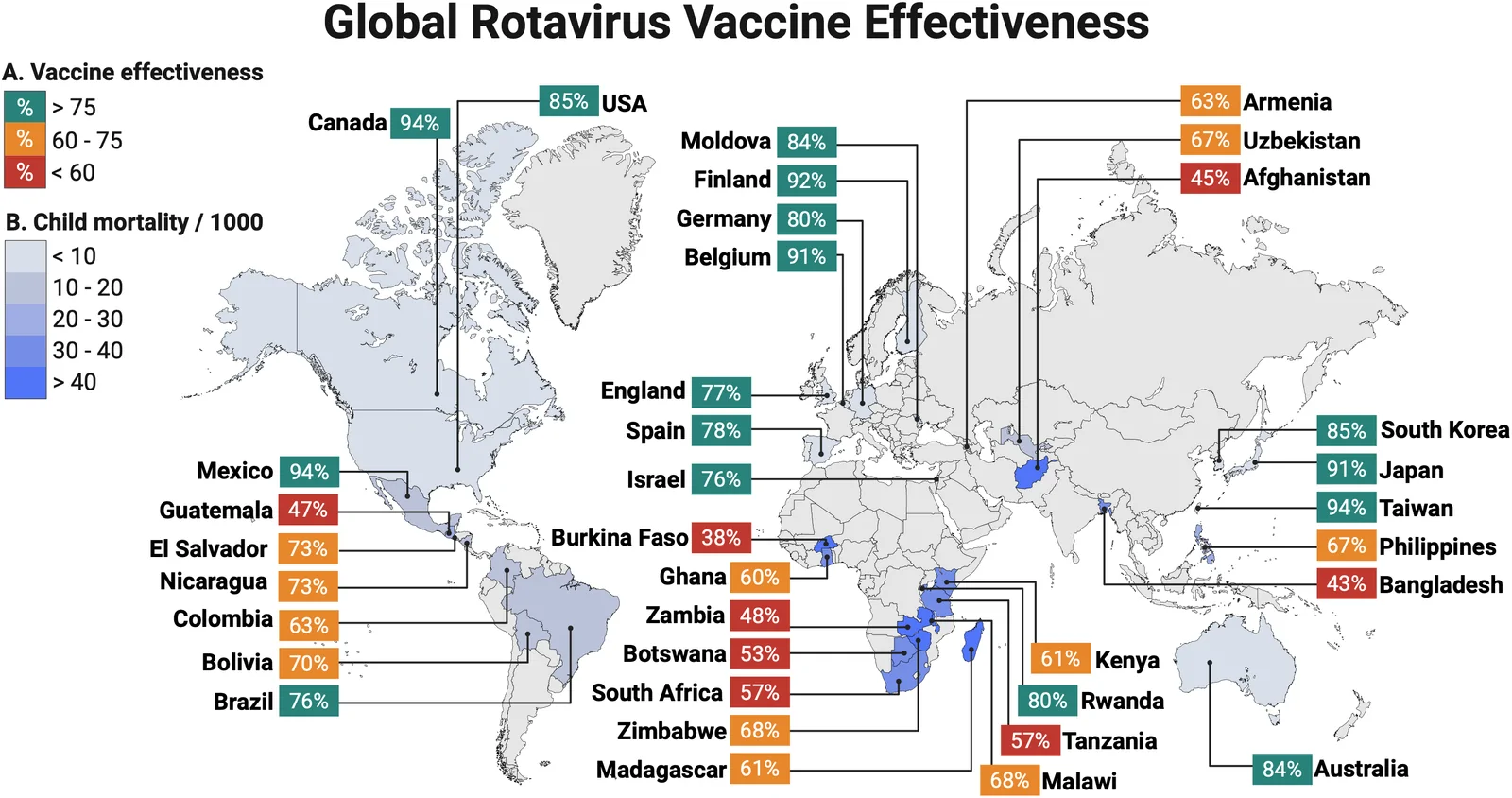
Global distribution of observed rotavirus vaccine effectiveness. Country-specific vaccine effectiveness estimates were obtained from Prunas and colleagues [5]. Only post-licensure case-control studies evaluating Rotarix or RotaTeq vaccination were included. Studies used laboratory-confirmed rotavirus hospitalization as the study endpoint, and derived vaccine effectiveness estimates from odds ratios. For child mortality, the most recent estimate of the under-5 mortality rate (deaths per 1,000 live births) for countries with available vaccine effectiveness data was included [6]. Created with BioRender.com.

Variation in vaccine effectiveness reflects a complex interplay of biological and environmental factors. Differences in maternal antibody levels, infant gut health, nutritional status, interference by co-administered vaccines, early natural exposure to rotavirus during infancy, and exposure to a wide range of other pathogens can all influence how effectively the rotavirus vaccine stimulates an immune response. Host genetic polymorphisms, such as variations in histo-blood group antigens (HBGAs), have also been shown to play a role in modulating vaccine effectiveness across populations. Variation in circulating strains of rotavirus has been considered as a reason for vaccine disparity, with higher diversity of rotavirus strains in LMICs [6]. However, strain diversity is unlikely to be the primary explanation as vaccine effectiveness remains lower in LMICs even against well-matched strains. Overall, the absence of well-defined immune correlates of protection has limited our ability to predict vaccine performance and identify the mechanisms underlying reduced vaccine effectiveness across different populations [7]. Understanding why rotavirus vaccine effectiveness varies across the globe is essential not only for improving current vaccination strategies, but also for guiding the development of next-generation vaccines and public health interventions aimed at reducing inequities in child health outcomes.

This review focuses primarily on the two most widely studied oral rotavirus vaccines, Rotarix and RotaTeq, as these account for the majority of the published literature investigating the factors underlying variation in vaccine effectiveness. We examine the main factors contributing to variable vaccine effectiveness, synthesize the current evidence, and consider what interventions and strategies may ultimately bridge the divide between vaccine performance in HICs and LMICs. Better understanding of these factors has the potential to reduce the disproportionate rotavirus mortality burden borne by the world’s most vulnerable children.

## 2. Maternal antibodies

Maternal antibodies are transferred to infants transplacentally during pregnancy and postnatally via breast milk. These antibodies provide critical early-life protection against pathogens, including rotavirus, but can also reduce the effectiveness of rotavirus vaccine [8,9]. Live attenuated oral vaccines rely on replication of the vaccine in the infant’s gut to stimulate a protective immune response. Maternal antibodies can block rotavirus vaccine replication, as evidenced by the correlation between high maternal antibody titers and low vaccine shedding in human infants [10,11]. This has also been demonstrated in a mouse model of neonatal rotavirus vaccination, whereby vaccine clearance by maternal antibodies was identified as the major mechanism of interference [12].

Maternal antibody interference with rotavirus vaccination has been observed in both HICs and LMICs [10,13,14]. In LMICs, high rates of natural rotavirus exposure mean that women of childbearing age typically accumulate higher titers of rotavirus-specific antibodies through repeated natural infections [15,16]. During pregnancy and breastfeeding it follows that more maternal antibodies will be delivered to infants in LMICs. Higher and more sustained rates of breastfeeding in these regions amplify this difference [9]. This is expected to produce markedly higher circulating IgG levels and higher mucosal IgA levels in infants at the time of vaccination in LMICs compared to levels present in infants in HICs. This will disproportionately reduce the effectiveness of rotavirus vaccination in LMICs. The very populations that most need effective vaccination are the same populations in which maternal antibody interference is most severe. However, maternal antibody titers alone do not fully explain the disparity in vaccine effectiveness. For example, Parker and colleagues reported no association between maternal antibody titers and rotavirus vaccine shedding in a UK cohort despite maternal antibody concentrations being comparable to those observed in India in maternal serum, and comparable to IgA in breast milk in Malawi [11].

The relative roles of interference mediated by transplacental IgG compared to breast milk IgA are uncertain. Some clinical studies have demonstrated a robust association between breast milk IgA titers and vaccine failure [14], but withholding breast milk at the time of vaccination has failed to improve immunogenicity [9]. This indicates that breast milk antibody interference does not represent a readily modifiable determinant of reduced vaccine effectiveness. Other studies have shown correlation between maternal IgG and vaccine effectiveness, suggesting that as transplacental IgG wanes in infants, additional booster doses may be a valuable future vaccine strategy [17].

## 3. Malnutrition, the microbiome and environmental enteric dysfunction

Poor infant nutritional status can substantially lower vaccine effectiveness in children [18]. A recent analysis of data from 19 countries showed that vaccine effectiveness is reduced in children with stunting, a marker of chronic malnutrition, compared with non-stunted peers [19]. Notably, acute malnutrition at the time of vaccine administration did not have a marked impact on vaccine effectiveness [19]. Micronutrients have also been explored as potential modifiers of oral vaccine responses. Although vitamin A is important for child health and immune function, deficiency has not shown a clear association with rotavirus vaccine responses [20]. In addition, despite the well-established role of zinc in immune function, daily zinc supplementation or co-administration with the vaccine has failed to increase seroconversion in infants [20–22].

Given the strong influence of diet on the intestinal microbiome, malnutrition is expected to substantially alter microbial composition. Several investigations have linked infant gut microbiota composition to rotavirus vaccine immunogenicity, though findings remain geographically inconsistent. While studies in Ghana, Pakistan and the US have identified specific bacterial species that correlate with seroconversion, studies in India, Malawi, Nicaragua and the UK have not [11,23–25]. These inconsistencies may reflect differences in many factors, including mode of birth, antibiotic exposure, maternal diet, sanitation, and breastfeeding practices, any of which could independently shape microbiota diversity. Microbiome diversity is another measure with which seroconversion can be evaluated, but again studies do not reach a consensus. Despite this, modifying the microbiome is still an easily implementable strategy that is being considered to improve vaccine take. Two independent studies in Finland and India have reported a modest increase in the immune response to rotavirus vaccine following supplementation of *Lactobacillus casei* GG and *Lactobacillus rhamnosus* GG, respectively [21,26].

Malnutrition and microbiome perturbations are closely intertwined with environmental enteric dysfunction (EED), an inflammatory disorder of the small intestine. EED is characterized by villous blunting, crypt hyperplasia, lymphocyte infiltration, and increased intestinal permeability, which together compromise nutrient absorption and sustain a chronically inflamed gut environment. Since small intestine biopsy is the only definitive gold standard diagnostic test, EED is difficult to diagnose. Noninvasive markers, including fecal myeloperoxidase, alpha-1-antitrypsin, calprotectin, I-FABP, plasma CRP, and alpha-1-acid glycoprotein, have been used to assess EED burden in multiple studies. Cohort studies in Bangladesh, Nicaragua, and Pakistan have consistently linked elevated EED biomarkers to reduced rotavirus vaccine seroconversion [27–29]. However, trials in Southern Africa and India found little or no association between most EED biomarkers and oral rotavirus vaccine responses, with some biomarkers even showing a weak positive relationship with seroconversion [11,30,31]. Many of these EED biomarkers are non-specific, and while current evidence supports an association between EED and impaired vaccine immune responses, this is not a definitively established independent causal role. Collectively, these findings highlight the complex interplay between malnutrition, microbiome disruption, and EED, and underscore the need for integrated nutritional, sanitary, and microbiome-targeted strategies to improve rotavirus vaccine immunogenicity in low-income settings.

## 4. Concurrent exposures: Vaccines and pathogens

Simultaneous administration of oral rotavirus vaccines and oral poliovirus vaccines (OPVs) has been shown to negatively interfere with rotavirus vaccine-induced immunity in multiple studies [32]. OPV has been widely replaced with the inactivated poliovirus vaccine (IPV) in HICs, but although many LMICs now use IPV in routine vaccine schedules, OPV remains highly effective in outbreak situations and has advantages over IPV in LMICs, including lower costs, easier implementation, and the ability to generate a mucosal immune response that can reduce transmission. As poliovirus cases predominantly occur in areas with limited public health infrastructure, this explains how the socioeconomic wealth of a region and rotavirus vaccine effectiveness can be connected. While the exact mechanism of OPV-mediated interference is unclear, it has been proposed that OPV replication induces a more rapid and robust immune response than the rotavirus vaccine, hence interfering with rotavirus vaccine replication [33]. Co-administration of oral rotavirus vaccines and OPV should be avoided by careful scheduling, although the minimum separation time between vaccines is not known. Furthermore, implementing staggered vaccination schedules may be operationally challenging, particularly in LMICs, where immunization programs rely on simple, standardized schedules to maximize vaccine coverage and minimize missed vaccination opportunities.

Natural infections by other enteric viruses at the time of rotavirus vaccination are common and less amenable to control than OPV-associated interference. Many enteric viruses circulate in infants, and co-infections with non-polio enteroviruses and novel cosaviruses have been shown to reduce vaccine effectiveness [34,35]. Increased bacteriophage richness has also been identified in non-seroconverting infants [35]. The impact of pathogenic bacterial infections at the time of vaccination is less clear, but understanding the role of the microbiome in vaccine effectiveness is an important area of research as considered above.

In addition to concurrent exposures to pathogens around the time of rotavirus vaccination, gastrointestinal infections pre- and post-vaccination can have confounding effects on perceived vaccine effectiveness. Early natural infection with rotavirus induces rotavirus-specific IgA prior to the first vaccine dose in up to 26% of infants in LMICs [13,36], whereas baseline seropositivity is typically under 5% in HICs [36]. Consequently, if vaccine performance is evaluated by fold-change seroconversion, this artificially reduces the effectiveness estimates in regions with more early transmission. Similarly, infections with non-rotavirus enteropathogens after vaccination may cause rotavirus vaccine effectiveness to be underestimated by artificially increasing the number of suspected vaccine failures. Several studies have identified rotavirus in combination with different enteric viruses or bacteria in cases of severe gastroenteritis post-vaccination [37,38]. It is possible rotavirus is not the primary cause of disease in these instances, but each gastroenteritis case would still be recorded as a vaccine failure. With the incidence of infectious gastroenteritis higher in LMICs, this will disproportionately affect these regions.

## 5. Human genetic polymorphisms

Variations in the host genome that influence the replication of live attenuated rotavirus vaccines have the potential to alter vaccine effectiveness. HBGAs are the most well-characterized host genetic factors influencing rotavirus replication. These antigens are secreted onto mucosal surfaces and are important for attachment of multiple rotavirus strains to the intestinal epithelia. HBGA secretion requires functional activity of fucosyltransferase 2 (FUT2). Several known polymorphisms can render FUT2 non-functional, resulting in a ‘non-secretor’ phenotype; for example, the most common polymorphism (G428A) introduces a premature stop codon. Multiple studies have demonstrated that non-secretors have significantly reduced antibody responses to rotavirus vaccination, with this being most pronounced for vaccines containing the P[8] outer capsid antigen [39]. The frequency of non-secretor polymorphisms varies among different populations, which may contribute to the reduced vaccine effectiveness observed in particular geographic areas. However, there is currently no clear evidence that the frequency of non-secretors in a population is associated with socioeconomic status.

The influence of fucosyltransferase 3 (FUT3) polymorphisms, which determine Lewis antigen expression, has also been considered in the context of rotavirus vaccine effectiveness. FUT3 adds an additional fucose to HBGAs, which in non-secretors generates the Lewis A antigen, and in secretors the Lewis B antigen. However, studies examining how FUT3 influences rotavirus vaccine take have yielded mixed outcomes, suggesting it is not a major factor [40]. Ultimately, evaluating the impact of non-secretor status and Lewis A phenotype on vaccine-induced protection from infection can be challenging; these infants are less susceptible to infection by many rotavirus strains, thus fewer post-vaccine infections are detected irrespective of vaccine effectiveness. In addition to the FUT2 and FUT3 status of an infant, maternal HBGA phenotypes have also been shown to impact infant vaccine take. FUT2- or FUT3-positive infants with FUT2- or FUT3-negative mothers are more likely to seroconvert [41]. The reasons for this are likely multifactorial; for example, fucosylated human milk oligosaccharides in breast milk can influence infant gut microbiota and may also act as decoys that interfere with the vaccine [39,41].

Further genetic factors associated with immune responses to rotavirus vaccines include variation in pattern recognition receptors of the TLR family [42], although it is not clear if this could be linked to geographical variation. A study assessing the full spectrum of genetic determinants influencing seroconversion to rotavirus vaccination is currently underway in a Vietnamese cohort, and is expected to provide additional insights [43].

## 6. Summary

The factors underlying rotavirus vaccine underperformance in LMICs are interconnected and geographically variable. Factors such as maternal antibody interference and OPV co-administration are relevant across all geographies but have a greater impact in LMICs due to higher endemic exposure to pathogens and continued OPV use. In contrast, the negative influence of EED on vaccine effectiveness is limited to regions where sanitation and nutrition are sub-optimal. However, no single factor acts independently. Maternal antibodies, malnutrition, and enteric pathogen burden each shape the infant gut environment and together create conditions that will limit oral vaccine replication. The gut microbiome can also differ between vaccine responders and non-responders in different populations, and no individual intervention is likely to be universally effective. Meaningful progress will require context-specific combinations of approaches, including optimizing vaccine schedules to reduce OPV and maternal antibody interference, and integrating vaccination with nutritional or probiotic support. Neonatal rotavirus vaccines such as RV3-BB may help circumvent several of the LMIC-specific barriers discussed above [44]. This is because neonatal vaccines are subjected to less interference from immunity to early-life co-infections and OPV, plus reduced interference from maternal antibodies as these vaccines are based on strains that rarely circulate in mothers. Novel parenteral vaccine platforms could also overcome some of these challenges, but identifying the optimal vaccine antigens and eliciting mucosal as well as systemic immunity will be critical. This is evidenced by the Phase 3 clinical trial of the parenteral trivalent subunit vaccine TV P2-VP8, which was found to be inferior to Rotarix in preventing severe rotavirus gastroenteritis [45]. Ultimately, vaccination remains the most pragmatic approach to reducing childhood rotavirus-induced disease, and fully characterizing the regional and biological determinants of oral vaccine underperformance is essential for maximizing the impact of existing vaccines and for guiding development of next-generation vaccine strategies (Fig 2).

**Fig 2:**
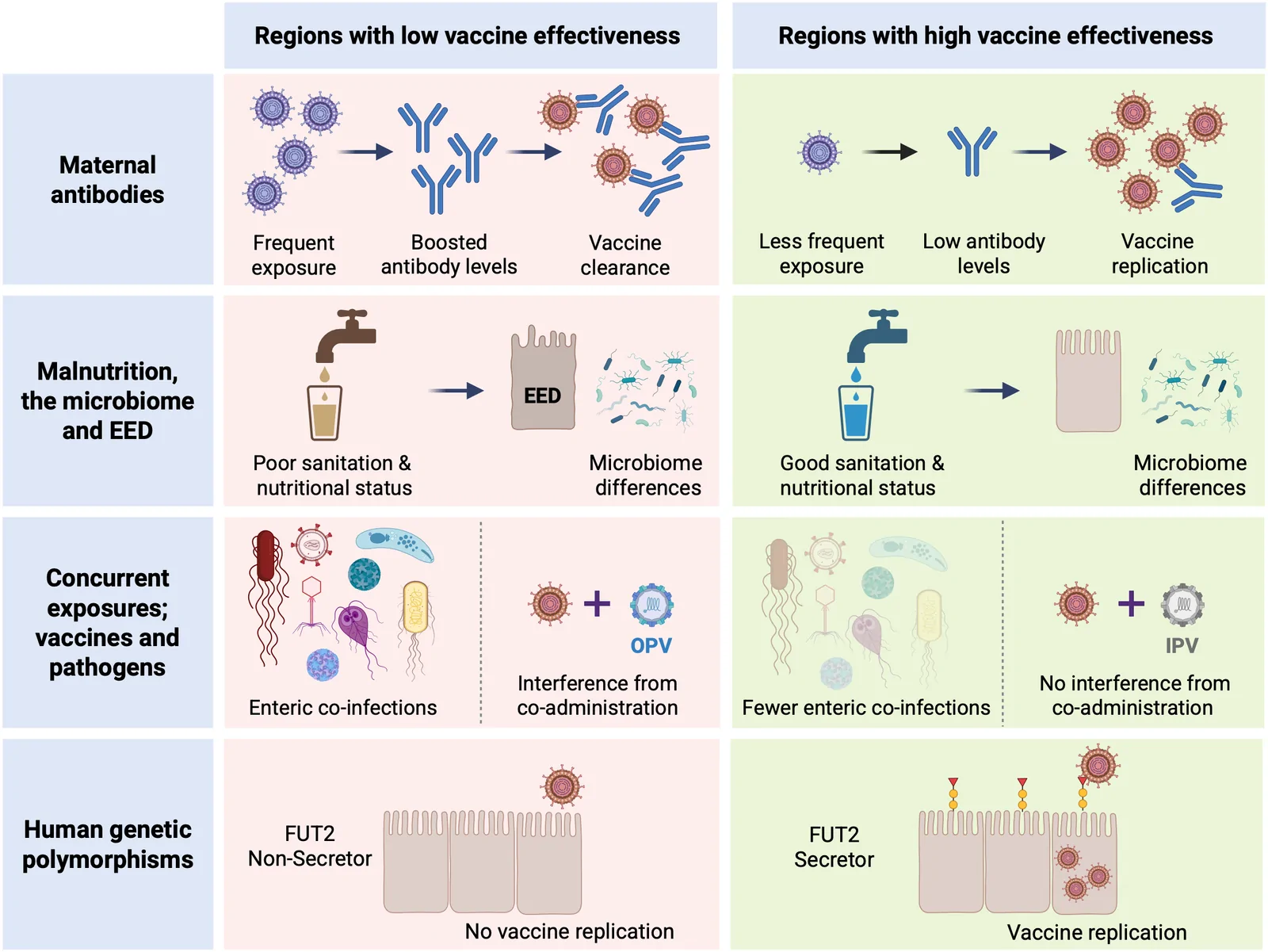
Major factors contributing to differences in rotavirus vaccine effectiveness. **(1) Maternal antibodies:** In regions with low vaccine effectiveness, frequent natural rotavirus exposure results in higher maternal rotavirus-specific antibody titers, which are transferred to infants and neutralize vaccine virus, limiting replication. In regions with high vaccine effectiveness, lower maternal antibody levels permit greater vaccine virus replication and stronger immune responses. **(2) Malnutrition, the microbiome and EED:** Poor sanitation and nutritional status in regions with low vaccine effectiveness contribute to environmental enteric dysfunction (EED) and altered gut microbial communities, which may impair immune responses to oral rotavirus vaccines. **(3) Concurrent exposures; vaccines and pathogens:** Regions with low vaccine effectiveness experience higher rates of enteric co-infections and use of the oral poliovirus vaccine (OPV), whereas high vaccine effectiveness regions generally have a lower pathogen burden and use the inactivated poliovirus vaccine (IPV). **(4) Human genetic polymorphisms:** Population differences in histo-blood group antigen expression, including fucosyltransferase 2 (FUT2) secretor status, may influence rotavirus vaccine uptake, replication, and effectiveness. Created with BioRender.com.

## Abbreviations

EED: environmental enteric dysfunction
HBGA: histo-blood group antigen
HICs: high-income countries
IPV: inactivated poliovirus vaccine
LMICs: low- and middle-income countries
OPV: oral poliovirus vaccine

## References

[1] Du Y, Chen C, Zhang X, Yan D, Jiang D, Liu X, et al. Global burden and trends of rotavirus infection-associated deaths from 1990 to 2019: An observational trend study. Virol J. 2022;19(1):166. doi: 10.1186/s12985-022-01898-9 36266651 PMC9585833

[2] International Vaccine Access Center (IVAC), Johns Hopkins Bloomberg School of Public Health. In: VIEW-hub [Internet]. 29 May 2026. Available from: https://www.view-hub.org

[3] Burnett E, Parashar UD, Tate JE. Real-world effectiveness of rotavirus vaccines, 2006-19: A literature review and meta-analysis. Lancet Glob Health. 2020;8(9):e1195–202. doi: 10.1016/S2214-109X(20)30262-X 32827481 PMC8097518

[4] Jonesteller CL, Burnett E, Yen C, Tate JE, Parashar UD. Effectiveness of rotavirus vaccination: A systematic review of the first decade of global postlicensure data, 2006–2016. Clinical Infectious Diseases. 2017;65:840–50. doi: 10.1093/cid/cix369 28444323

[5] Prunas O, Asare EO, Sajewski E, Li Y, Pithawala Z, Weinberger DM, et al. Global estimates of rotavirus vaccine efficacy and effectiveness: A rapid review and meta-regression analysis. EClinicalMedicine. 2025;81:103122. doi: 10.1016/j.eclinm.2025.103122 40115174 PMC11925534

[6] Amin AB, Tate JE, Waller LA, Lash TL, Lopman BA. Monovalent rotavirus vaccine efficacy against different rotavirus genotypes: A pooled analysis of phase II and III trial data. Clin Infect Dis. 2023;76(3):e1150–6. doi: 10.1093/cid/ciac699 36031386 PMC10169401

[7] Clarke E, Desselberger U. Correlates of protection against human rotavirus disease and the factors influencing protection in low-income settings. Mucosal Immunol. 2015;8(1):1–17. doi: 10.1038/mi.2014.114 25465100

[8] Desselberger U. Differences of rotavirus vaccine effectiveness by country: Likely causes and contributing factors. Pathogens. 2017;6(4):65. doi: 10.3390/pathogens6040065 29231855 PMC5750589

[9] Otero CE, Langel SN, Blasi M, Permar SR. Maternal antibody interference contributes to reduced rotavirus vaccine efficacy in developing countries. PLoS Pathog. 2020;16(11):e1009010. doi: 10.1371/journal.ppat.1009010 33211756 PMC7676686

[10] Burke RM, Payne DC, McNeal M, Conrey SC, Burrell AR, Mattison CP, et al. Correlates of rotavirus vaccine shedding and seroconversion in a US cohort of healthy infants. J Infect Dis. 2024;230(3):754–62. doi: 10.1093/infdis/jiae055 38330312 PMC12093357

[11] Parker EPK, Bronowski C, Sindhu KNC, Babji S, Benny B, Carmona-Vicente N, et al. Impact of maternal antibodies and microbiota development on the immunogenicity of oral rotavirus vaccine in African, Indian, and European infants. Nat Commun. 2021;12(1):7288. doi: 10.1038/s41467-021-27074-1 34911947 PMC8674366

[12] Chandler TL, Woodyear S, Chen V, Lonergan TM, Baker N, Harcourt K, et al. Mechanisms of maternal antibody interference with rotavirus vaccination. EMBO J. 2025;44(22):6343–67. doi: 10.1038/s44318-025-00582-2 41087582 PMC12623505

[13] Becker-Dreps S, Vilchez S, Velasquez D, Moon S-S, Hudgens MG, Zambrana LE, et al. Rotavirus-specific IgG antibodies from mothers’ serum may inhibit infant immune responses to the pentavalent rotavirus vaccine. Pediatr Infect Dis J. 2015;34(1):115–6. doi: 10.1097/INF.0000000000000481 25741808 PMC4352718

[14] Chilengi R, Simuyandi M, Beach L, Mwila K, Becker-Dreps S, Emperador DM, et al. Association of maternal immunity with rotavirus vaccine immunogenicity in Zambian infants. PLoS One. 2016;11(3):e0150100. doi: 10.1371/journal.pone.0150100 26974432 PMC4790930

[15] Moon S-S, Tate JE, Ray P, Dennehy PH, Archary D, Coutsoudis A, et al. Differential profiles and inhibitory effect on rotavirus vaccines of nonantibody components in breast milk from mothers in developing and developed countries. Pediatr Infect Dis J. 2013;32(8):863–70. doi: 10.1097/INF.0b013e318290646d 23584581 PMC4610365

[16] Novak D, Svennerholm A-M. A comparison of seasonal variations in rotavirus antibodies in the breast milk of Swedish and Bangladeshi mothers. Acta Paediatr. 2015;104(3):247–51. doi: 10.1111/apa.12841 25355609

[17] Mandolo J, Mulira L, Moyo M, Mvula M, Mtonga F, Henrion MYR, et al. Maternal-infant rotavirus-specific antibody kinetics to inform timing of vaccine boosting in Malawi: An observational study. PLoS Med. 2025;22(9):e1004734. doi: 10.1371/journal.pmed.1004734 40938968 PMC12445545

[18] Burnett E, Parashar UD, Tate JE. Rotavirus infection, illness, and vaccine performance in malnourished children: A review of the literature. Pediatr Infect Dis J. 2021;40(10):930–6. doi: 10.1097/INF.0000000000003206 34117200 PMC8489158

[19] Burnett E, Ticklay I, Umana J, Mandomando I, Robinson AL, Omore R, et al. Oral rotavirus vaccine effectiveness among malnourished children in 19 countries: Findings from the MNSSTER-V project. J Pediatr Gastroenterol Nutr. 2026;82(3):790–800. doi: 10.1002/jpn3.70322 41486460 PMC12794440

[20] Church JA, Parker EP, Kirkpatrick BD, Grassly NC, Prendergast AJ. Interventions to improve oral vaccine performance: A systematic review and meta-analysis. Lancet Infect Dis. 2019;19(2):203–14. doi: 10.1016/S1473-3099(18)30602-9 30712836 PMC6353819

[21] Lazarus RP, John J, Shanmugasundaram E, Rajan AK, Thiagarajan S, Giri S, et al. The effect of probiotics and zinc supplementation on the immune response to oral rotavirus vaccine: A randomized, factorial design, placebo-controlled study among Indian infants. Vaccine. 2018;36(2):273–9. doi: 10.1016/j.vaccine.2017.07.116 28874323 PMC12001858

[22] Isanaka S, Garba S, Plikaytis B, Malone McNeal M, Guindo O, Langendorf C, et al. Immunogenicity of an oral rotavirus vaccine administered with prenatal nutritional support in Niger: A cluster randomized clinical trial. PLoS Med. 2021;18(8):e1003720. doi: 10.1371/journal.pmed.1003720 34375336 PMC8354620

[23] Harris VC, Armah G, Fuentes S, Korpela KE, Parashar U, Victor JC, et al. Significant correlation between the infant gut microbiome and rotavirus vaccine response in rural Ghana. J Infect Dis. 2017;215(1):34–41. doi: 10.1093/infdis/jiw518 27803175 PMC5225256

[24] Fix J, Chandrashekhar K, Perez J, Bucardo F, Hudgens MG, Yuan L, et al. Association between gut microbiome composition and rotavirus vaccine response among Nicaraguan infants. Am J Trop Med Hyg. 2020;102(1):213–9. doi: 10.4269/ajtmh.19-0355 31802728 PMC6947780

[25] Harris V, Ali A, Fuentes S, Korpela K, Kazi M, Tate J, et al. Rotavirus vaccine response correlates with the infant gut microbiota composition in Pakistan. Gut Microbes. 2018;9(2):93–101. doi: 10.1080/19490976.2017.1376162 28891751 PMC5989807

[26] Isolauri E, Joensuu J, Suomalainen H, Luomala M, Vesikari T. Improved immunogenicity of oral D x RRV reassortant rotavirus vaccine by *Lactobacillus casei* GG. Vaccine. 1995;13(3):310–2. doi: 10.1016/0264-410x(95)93319-5 7631519

[27] Ahmed S, Iqbal J, Sadiq K, Umrani F, Rizvi A, Kabir F, et al. Association of anti-rotavirus IgA seroconversion with growth, environmental enteric dysfunction and enteropathogens in rural Pakistani infants. Vaccine. 2022;40(25):3444–51. doi: 10.1016/j.vaccine.2022.04.032 35534310 PMC9168439

[28] Naylor C, Lu M, Haque R, Mondal D, Buonomo E, Nayak U, et al. Environmental enteropathy, oral vaccine failure and growth faltering in infants in Bangladesh. EBioMedicine. 2015;2(11):1759–66. doi: 10.1016/j.ebiom.2015.09.036 26870801 PMC4740306

[29] Becker-Dreps S, Vilchez S, Bucardo F, Twitchell E, Choi WS, Hudgens MG, et al. The association between fecal biomarkers of environmental enteropathy and rotavirus vaccine response in Nicaraguan infants. Pediatr Infect Dis J. 2017;36(4):412–6. doi: 10.1097/INF.0000000000001457 27977553

[30] Mwila-Kazimbaya K, Bosomprah S, Chilyabanyama ON, Chisenga CC, Chibuye M, Laban NM, et al. Association of biomarkers of enteric dysfunction, systemic inflammation, and growth hormone resistance with seroconversion to oral rotavirus vaccine: A lasso for inference approach. PLoS One. 2023;18(11):e0293101. doi: 10.1371/journal.pone.0293101 37976323 PMC10656027

[31] Church JA, Rukobo S, Govha M, Gough EK, Chasekwa B, Lee B, et al. Associations between biomarkers of environmental enteric dysfunction and oral rotavirus vaccine immunogenicity in rural Zimbabwean infants. EClinicalMedicine. 2021;41:101173. doi: 10.1016/j.eclinm.2021.101173 34825149 PMC8605235

[32] Baker JM, Tate JE, Leon J, Haber MJ, Lopman BA. Antirotavirus IgA seroconversion rates in children who receive concomitant oral poliovirus vaccine: A secondary, pooled analysis of Phase II and III trial data from 33 countries. PLoS Med. 2019;16(12):e1003005. doi: 10.1371/journal.pmed.1003005 31887139 PMC6936798

[33] Emperador DM, Velasquez DE, Estivariz CF, Lopman B, Jiang B, Parashar U. Interference of monovalent, bivalent, and trivalent oral poliovirus vaccines on monovalent rotavirus vaccine immunogenicity in rural Bangladesh. Clin Infect Dis. 2016;62:150–6. doi: 10.1093/cid/civ807 PMC4755336 26349548

[34] Taniuchi M, Platts-Mills JA, Begum S, Uddin MJ, Sobuz SU, Liu J, et al. Impact of enterovirus and other enteric pathogens on oral polio and rotavirus vaccine performance in Bangladeshi infants. Vaccine. 2016;34(27):3068–75. doi: 10.1016/j.vaccine.2016.04.080 27154394 PMC4912219

[35] Kim AH, Armah G, Dennis F, Wang L, Rodgers R, Droit L, et al. Enteric virome negatively affects seroconversion following oral rotavirus vaccination in a longitudinally sampled cohort of Ghanaian infants. Cell Host Microbe. 2022;30(1):110-123.e5. doi: 10.1016/j.chom.2021.12.002 34932985 PMC8763403

[36] Cunliffe N, Zaman K, Rodrigo C, Debrus S, Benninghoff B, Pemmaraju Venkata S, et al. Early exposure of infants to natural rotavirus infection: a review of studies with human rotavirus vaccine RIX4414. BMC Pediatr. 2014;14:295. doi: 10.1186/s12887-014-0295-2 25433534 PMC4261882

[37] Praharaj I, Platts-Mills JA, Taneja S, Antony K, Yuhas K, Flores J, et al. Diarrheal etiology and impact of coinfections on rotavirus vaccine efficacy estimates in a clinical trial of a monovalent human-bovine (116E) oral rotavirus vaccine, Rotavac, India. Clin Infect Dis. 2019;69(2):243–50. doi: 10.1093/cid/ciy896 30335135 PMC6603264

[38] Simsek C, Bloemen M, Jansen D, Beller L, Descheemaeker P, Reynders M, et al. High prevalence of coinfecting enteropathogens in suspected rotavirus vaccine breakthrough cases. J Clin Microbiol. 2021;59(12):e0123621. doi: 10.1128/JCM.01236-21 34586890 PMC8601229

[39] Sharma S, Nordgren J. Effect of infant and maternal secretor status on rotavirus vaccine take-an overview. Viruses. 2021;13(6):1144. doi: 10.3390/v13061144 34198720 PMC8232156

[40] Sharma S, Hagbom M, Svensson L, Nordgren J. The impact of human genetic polymorphisms on rotavirus susceptibility, epidemiology, and vaccine take. Viruses. 2020;12:324. doi: 10.3390/v12030324 PMC7150750 32192193

[41] Pun J, Evans C, Chasekwa B, Church JA, Gough E, Mutasa K, et al. Associations between histo-blood group antigen status in mother-infant dyads and infant oral rotavirus vaccine immunogenicity in rural Zimbabwe. J Infect Dis. 2025;231(1):e225–33. doi: 10.1093/infdis/jiae456 39352457 PMC11793023

[42] Miya TV, Groome MJ, de Assis Rosa D. TLR genetic variation is associated with Rotavirus-specific IgA seroconversion in South African Black infants after two doses of Rotarix vaccine. Vaccine. 2021;39(48):7028–35. doi: 10.1016/j.vaccine.2021.10.051 34740476 PMC8678908

[43] de Alwis R, My Phuc T, Yu Hang Bai B, Le Thi Quynh N, Thi Thanh Tam P, Thi Ngoc Dung T, et al. The influence of human genetic variation on early transcriptional responses and protective immunity following immunization with Rotarix vaccine in infants in Ho Chi Minh City in Vietnam: A study protocol for an open single-arm interventional trial. Wellcome Open Res. 2020;5:246. doi: 10.12688/wellcomeopenres.16090.1

[44] Bines JE, At Thobari J, Satria CD, Handley A, Watts E, Cowley D, et al. Human neonatal rotavirus vaccine (RV3-BB) to target rotavirus from birth. N Engl J Med. 2018;378(8):719–30. doi: 10.1056/NEJMoa1706804 29466164 PMC5774175

[45] Tewari T, Armah G, Cunliffe NA, Chisenga CC, Witte D, Kukula V, et al. Safety, immunogenicity, and relative efficacy of a parenteral trivalent rotavirus subunit vaccine candidate (TV P2-VP8) in healthy Ghanaian, Malawian, and Zambian infants. Vaccine. 2026;70:128019. doi: 10.1016/j.vaccine.2025.128019 41319434

